# PDT-YOLO: A Roadside Object-Detection Algorithm for Multiscale and Occluded Targets

**DOI:** 10.3390/s24072302

**Published:** 2024-04-04

**Authors:** Ruoying Liu, Miaohua Huang, Liangzi Wang, Chengcheng Bi, Ye Tao

**Affiliations:** 1 Hubei Key Laboratory of Advanced Technology for Automotive Components, Wuhan University of Technology, Wuhan 430070, China; lrywhlg@whut.edu.cn (R.L.); wliangzi@whut.edu.cn (L.W.); bcc_@whut.edu.cn (C.B.); tao-ye@whut.edu.cn (Y.T.); 2 Hubei Collaborative Innovation Center for Automotive Components Technology, Wuhan University of Technology, Wuhan 430070, China; 3 Hubei Research Center for New Energy & Intelligent Connected Vehicle, Wuhan 430070, China

**Keywords:** roadside perception, intra-scale feature interaction module, multi-scale efficient layer aggregation network, multi-attention mechanism, robot operating system, YOLOv7-tiny

## Abstract

To tackle the challenges of weak sensing capacity for multi-scale objects, high missed detection rates for occluded targets, and difficulties for model deployment in detection tasks of intelligent roadside perception systems, the PDT-YOLO algorithm based on YOLOv7-tiny is proposed. Firstly, we introduce the intra-scale feature interaction module (AIFI) and reconstruct the feature pyramid structure to enhance the detection accuracy of multi-scale targets. Secondly, a lightweight convolution module (GSConv) is introduced to construct a multi-scale efficient layer aggregation network module (ETG), enhancing the network feature extraction ability while maintaining weight. Thirdly, multi-attention mechanisms are integrated to optimize the feature expression ability of occluded targets in complex scenarios, Finally, Wise-IoU with a dynamic non-monotonic focusing mechanism improves the accuracy and generalization ability of model sensing. Compared with YOLOv7-tiny, PDT-YOLO on the DAIR-V2X-C dataset improves mAP50 and mAP50:95 by 4.6% and 12.8%, with a parameter count of 6.1 million; on the IVODC dataset by 15.7% and 11.1%. We deployed the PDT-YOLO in an actual traffic environment based on a robot operating system (ROS), with a detection frame rate of 90 FPS, which can meet the needs of roadside object detection and edge deployment in complex traffic scenes.

## 1. Introduction

The accurate assessment of the surrounding environment is a crucial task for intelligent autonomous vehicles to ensure secure movement [1]. However, the placement and perspective of onboard sensors contribute to the lack of global vision and limited remote sensing of autonomous vehicles [2]. Intelligent roadside sensing systems can be used to address the sensory limitations of autonomous vehicles in urban traffic scenarios by sharing information about pedestrians, vehicles, and traffic signs through wireless communication devices to provide enhanced environment awareness information for autonomous vehicles, decreasing the possibility of accidents and enhancing road traffic efficiency [3].

The current research on intelligent roadside sensing technologies can be classified as radar-based, camera-based, LiDAR-based, and multi-sensor fusion-based according to the type of sensor. Radar [4] is capable of obtaining data about moving objects within a specific region, although its precision in perception is limited. LiDAR [5,6,7] acquires point cloud data by utilizing laser scanning, which captures details regarding the target object’s position and dimensions. The method includes advantages such as high accuracy, wide sensing range, and immunity to light interference, but it is costly to deploy on the roadside. The multi-sensor fusion-based method [8,9,10] combines diverse data from several sensors to enhance the precision and reliability of the system. However, it involves issues such as time and space synchronization between multiple sensors as well as high computational complexity. Compared to other sensors, the camera-based method is low-cost, can obtain rich environmental information, and has real-time performance. 

The conventional image-based roadside object-detection algorithms consist of the optical flow method [11], inter-frame difference method [12], background subtraction method [13], etc. These algorithms rely mainly on hand-designed characteristics and classifiers driven by expert knowledge and experience. However, artificial design features contain only low-level information, so their expressive and descriptive capabilities are always limited, resulting in poor generalization.

Due to advancements in information technology, particularly convolutional neural networks, deep learning techniques have become extensively utilized in object identification tasks, such as R-CNN [14,15,16] series, FCOS [17] and SSD [18], YOLO [19,20,21,22] series, etc. These one-stage or two-stage methods achieve more robust and accurate object-detection tasks in various complex scenarios by training and learning features on large-scale datasets. In recent years, the YOLO series has undergone constant optimization, with significantly improved detection accuracy and speed, lower computational costs, and more excellent comprehensive performance [23]. Zhang et al. [24] used the Res3Unit structure and a label allocation module with a Gaussian receptive field to reconstruct YOLOv7, improving the receptive field for small targets and solving the problem of the missed detection rate. Huang et al. [25] proposed a model based on YOLOv5s called RD-YOLO, which replaced the original pyramid network through a broad-based characteristic pyramid network and integrated coordinate attention (CA) mechanism. Although roadside cameras have a wider and longer field of view, perception algorithms based on roadside cameras also have corresponding problems in complex traffic environments, such as missed detection of targets caused by occlusion between traffic participants in dense traffic and false detection of multi-scale targets.

Multi-scale context information is essential for targets with different scales. Context information of different scales can be concatenated to gain multi-scale information to improve the performance of detection. Deng et al. [26] proposed MS-OPN, a multi-scale object proposal network based on several intermediate feature maps, according to the certain scale ranges of different objects. Zeng et al. [27] proposed the atrous spatial pyramid pooling balanced feature pyramid network called ABFPN, a novel enhanced multiscale feature fusion method. To fully utilize context information, atrous convolution operators with varying dilation rates are used, and skip connections are applied to achieve sufficient feature fusions. Shen et al. [28] and Ju et al. [29] proposed a YOLOv3-based method in which a 4 times smaller detection branch is added and a feature map cropping module is introduced. Xu et al. [30] adopted a densely connected network to enhance the feature extraction capability of YOLOv3. Han et al. [31] proposed a multi-scale feature extraction module called LM-fem to enhance the multi-scale feature extraction capability and a new hybrid domain attention module called S-ECA relying on multi-scale contextual information. The above methods improve the model’s ability to extract multi-scale contextual information by optimizing the structure of the feature extraction network but inevitably increase the complexity of the network.

Occluded objects are characterized by a limited number of pixels, presenting incomplete features and being obscured by noise and background clutter. After successive down-sampling and pooling operations, part of the features will be lost. Several researchers have undertaken studies on that. Tian et al. [32] proposed a vehicle detection grammar to handle partial occlusion, including structure, deformation, and pairwise SVM grammars which captures rich information about the vehicle and occlusions. Whereas, dividing vehicles into semantic parts and then designing detection programs based on grammar models to perform vehicle detection and handle vehicle occlusion issues cannot ensure that the initial sampling network learns more suitable features for occlusion representation. Zhang et al. [33] proposed a Faster R-CNN-based method that employed a channel-wise attention mechanism to handle various occlusion patterns. Zhang et al. [34] proposed a detector based on Faster R-CNN integrated with a part-aware region proposal network to extract global and local visual information about vehicles. By generating partial and instance-level proposals and encoding different parts of one vehicle into a compositional proposal, the detector model reduces the impact of occlusion. Li et al. [35] proposed a detector based on YOLOv3 called YOLO-ACN with a channel attention module, realizing the cross-channel interaction without dimensionality reduction, which can pay more attention to the occluded objects. Song et al. [36] proposed a progressive refinement network called PRNet and PRNet++ with a dual-stream structure with occlusion loss and receptive field back-feed modules. While the aforementioned studies have made valuable contributions to occluded-object detection, they still exhibit certain limitations such as complex networks and slow inference speed. Our research aims to build upon these works and provide a lightweight and high-precision network architecture to address the challenges.

In summary, prior methodologies have demonstrated some efficacy in detecting multi-scale and occluded targets. Nonetheless, the intricacies of urban transportation environments, coupled with infrastructure limitations, necessitate a balance between accuracy, model weight, and computational efficiency in the deployment of roadside systems. Addressing these challenges, this article presents optimizations to the YOLOv7-tiny model and introduces the PDT-YOLO roadside object-detection algorithm. This article has carried out the following work:

(1) Revise the structure of the feature fusion layer and incorporate the AIFI module to enhance the precision of multi-scale object identification and reduce network parameters and model calculations.
(2) Develop a multi-scale feature extraction ETG module to replace the ELAN-T module in the head network to enhance the detection accuracy while maintaining the number of model parameters and computational complexity.
(3) Employ multi-attention mechanism modules to augment feature processing, mitigate intricate background interference, and amplify the expressive capacity of occluded objects.
(4) Utilize the WIoU to enhance the accuracy and generalization adaptability within the network.
(5) Implement perception algorithms on the roadside and perform real-time object detection and verification based on ROS (Robot Operating System).

## 2. Methodology

### 2.1. YOLOv7-Tiny Network

The YOLOv7 algorithm is a one-stage network released by Wang Chienyao’s team in 2022, which possesses the benefits of fast inference and high detection accuracy. YOLOv7-tiny is a lightweight YOLOv7 network suitable for edge GPUs, which mainly consists of two parts: the backbone network and the detection head network. The backbone network combines standard convolution, efficient layer aggregation network (ELAN-T), and maximum pooling convolution module (MPConv) to perform multiple feature extraction and scale transformation on the input, obtaining multi-scale feature information. The head network initially employs an enhanced spatial pyramid pooling module (SPPCSPC) to mitigate image distortion and eliminate redundant feature extraction issues. Additionally, it employs a feature pyramid framework to transfer and merge features. Ultimately, it uses standard convolution and the Detect module to obtain multiple prediction boxes and outputs predicted bounding box coordinate information, confidence, and class probability.

### 2.2. Feature Fusion Network Improvement

The detection of multi-scale and occluded objects poses a challenge in the field of computer vision. Due to the occluded object’s relatively weak features in the image, it may not be able to capture details and contextual information, resulting in inaccurate detection results. In the realm of roadside object-detection algorithms, there exists a dual imperative: the accurate identification of targets in complex road environments and the model size for the feasible deployment of edge devices on the roadside. 

The YOLOv7-tiny model incorporates the PANet [37] structure within its head component which achieves cross-level feature interaction and fusion by adding bottom-up enhancement routes and adaptive feature pooling operations. Such a design enables the effective utilization of semantic information across diverse levels, enhancing detection accuracy concerning multi-scale targets. When the input image size is set to 640 × 640, the backbone feature extraction network of the YOLOv7-tiny model generates feature maps sized 320 × 320, 160 × 160, 80 × 80, 40 × 40, and 20 × 20 through the ELAN-T module and the MP module.

**Figure 1:**
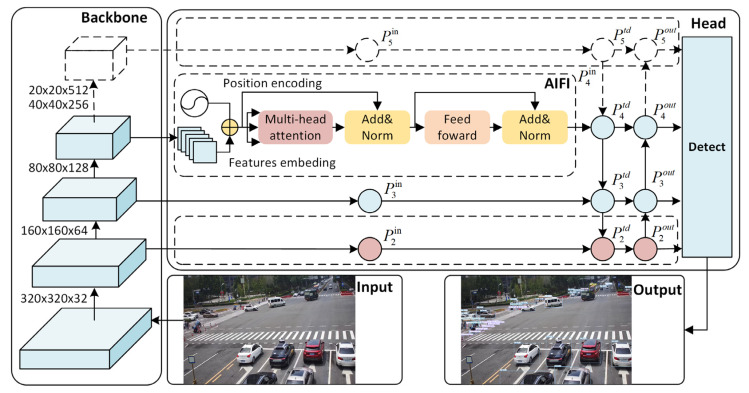
Structure of improved feature fusion layer.

Firstly, to improve the detection performance of small targets and reduce network parameters and model calculations, the processing of feature maps with a size of 160 × 160 is increased, while removing the top-level feature extraction layer of the backbone network which means feature maps with a size of 20 × 20 has been removed, as shown in Figure 1. Deeper feature maps with larger receptive fields are more suitable for detecting large objects, while low-level feature maps with smaller receptive fields are more suitable for detecting small objects. In essence, this article focuses on processing feature maps of 160 × 160, 80 × 80, and 40 × 40 to effectively combine contextual information for smaller objects and maintain a three-layer network structure. Considering the bottom-up path aggregation network and the operation of cross-scale connections, the operation process of the third layer node is as follows:

(1) $$P_{3}^{td}=\mathrm{Conv}\left(P_{3}^{in}+Resize\left(P_{4}^{td}\right)\right)$$

(2) $$P_{3}^{out}=Conv\left(P_{3}^{td}+\mathrm{Resize}\left(P_{2}^{out}\right)\right)$$

In the above equation: $P_{3}^{in}$ is the original input feature of the third layer, $P_{3}^{td}$ and $P_{4}^{td}$ are the intermediate feature levels of the third and fourth layers in the top-down path, $P_{2}^{out}$ and $P_{3}^{out}$ are the output features of the second and third layers in the bottom-up path.

Secondly, to add richer advanced semantic features, a transformer-based module called AIFI [38] is introduced. Transformer [39] is a deep learning model based on a self-attention mechanism, widely used in natural language processing tasks. The encoder module in a Transformer is composed of a stack of 6 identical layers, mainly including multi-head self-attention, pointwise feedforward, and normalization. Multi-head self-attention is a key component of Transformer which allows the model to jointly focus on information from different representation subspaces at different positions. The equations of the multi-head self-attention are detailed below:

(3) $$\mathrm{MultiHead}\left(Q,K,V\right)=\mathrm{Concat}\left({\mathrm{head}}_{1},\ldots ,head_{h}\right)W^{O}$$

(4) $${\mathrm{head}}_{i}=\mathrm{Attention}\left(QW_{i}^{Q},KW_{i}^{K},VW_{i}^{V}\right)$$

The query, key, and value matrix are the inputs of the attention mechanism. $W_{i}^{Q},\;W_{i}^{K},\;W_{i}^{V}$ represent the weight matrix of the linear transformation related to the attention head, projecting the input into different subspaces. $W^{O}$ is the final linear transformation to obtain the final output of multi-head self-attention. 

Pointwise feedforward introduces nonlinearity and allows the model to independently capture complex patterns in the data at each position. This enables the network to learn the complex relationships between different elements in the input sequence which helps in modeling and processing sequence data. The equation of the feedforward network is detailed below:

(5) $$\mathrm{FFN}\left(x\right)=\max\left(0,{\mathrm{xW}}_{1}+b_{1}\right)W_{2}+b_{2}$$

Among them, x represents the input, $W_{1}$, $b_{1}$, $W_{2}$ and $b_{2}$ are the weight matrix and bias terms of the first and second linear transformation. 

Lv et al. [38] introduced an efficient hybrid encoder based on the Transformer which converts multi-scale features into image feature sequences through intra-scale feature interaction (AIFI) and cross-scale feature fusion module (CCFM). The AIFI module orchestrates intra-scale interaction among high-level features through a self-attention mechanism, enabling the capture of relationships between conceptual entities within the image. This mechanism proves advantageous for subsequent modules tasked with object detection and recognition. The equations of the AIFI module are detailed below:

(6) $$Q=K=V=\mathrm{Flatten}\left(P_{4}\right)$$

(7) $$P_{4}^{\mathrm{in}}=\mathrm{Reshape}\left(\mathrm{FFN}\left(\mathrm{MultiHead}\left(Q,K,V\right)\right)\right)$$

In the above equation, the query, key, and value are the same and all come from the results of the flatten operation of the fourth layer. The flatten operation collapses the width and height dimensions of the input tensor into a single dimension while preserving the batch and channel dimensions. This paper uses the AIFI module instead of the SPPCSP structure to process the high-level feature maps of the model, reducing computational complexity and improving detection accuracy.

### 2.3. ETG Module

**Figure 2:**
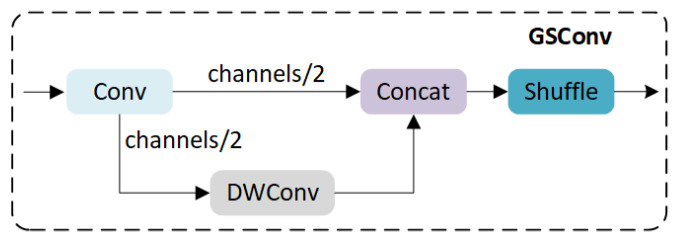
Structure of GSConv module.

To enhance the expression ability of image features while ensuring the number of parameters and detection speed of the algorithm model, this paper introduces the GSConv module and develops an ETG structure. GSConv is a lightweight convolution technique to reduce model weight while maintaining accuracy, proposed by Li et al. [40], which combines the advantages of a standard convolution module, a depth-wise separable convolution module, and a Shuffle. The GSConv structure is shown in Figure 2.

**Figure 3:**
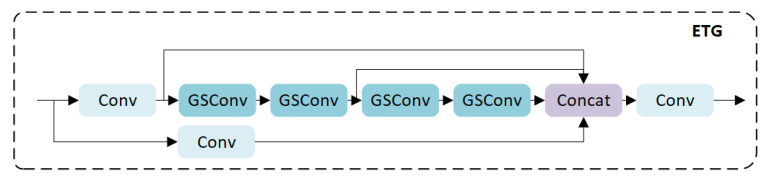
Structure of ETG module.

ELAN [41] is a strategy for designing gradient paths at the network level, which optimizes the gradient path length of the entire network by introducing a stacking structure in the calculation block. The main purpose of ELAN is to address the problem of deteriorating convergence of deep models during model expansion. The ETG structure is proposed based on the idea of ELAN and consists of four parallel branches. As shown in Figure 3, two branches use 1 × 1 convolution to preserve the texture and background features of the image, while the other two branches use two cascaded GSConv modules to improve feature fusion, accelerate network inference speed, and effectively reduce network complexity. 

### 2.4. Multi-Attention Mechanism Module

**Figure 4:**
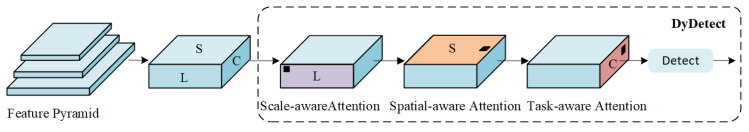
Structure of the DyDetect module.

To improve the expression ability of the detection head, a multi-attention mechanism called the DyHead [42] module is added to the original Detect module, which is optimized to DyDetect. The network structure diagram of DyDetect is shown in Figure 4. The DyHead module contains a scale-aware attention mechanism $\pi_{L}$, a spatial-aware attention mechanism $\pi_{S}$, and a task-aware attention mechanism $\pi_{C}$. The equations of DyHead are as follows:

(8) $$\pi_{L}\left(F\right)=HSigmoid\left(ReLU\left(Conv2d\left(AvgPool\left(F\right)\right)\right)\right)$$

(9) $$\left\{\begin{array}{c} \pi_{S}\left(F\right)=\sum_{F=level-1}^{level+1}DyConv\left(F\right)\cdot \pi_{L}\left(DyConv\left(F\right)\right)\\ DyConv\left(F\right)=DyDCNv2\left(F;mask\left(F\right);offset\left(F\right)\right) \end{array}\right.$$

(10) $$\pi_{C}\left(F\right)=max\left(FC\left(AvgPool\left(\pi_{S}\left(F\right)\right)\right)\right)$$

The scale-aware attention mechanism conducts average pooling on the input feature maps, resizing them to a 1 × 1 size. Through a convolutional operation to reduce the dimensionality of the feature map. Subsequently, the ReLU is applied to introduce non-linearity, facilitating the model in learning complex feature relationships. The use of the hard-sigmoid function aims to strike a balance between the model’s expressive power and training efficiency.

The spatial-aware attention mechanism calculates the mask and offset based on the input feature map (level), and then uses a dynamic convolutional network (DyDCNv2) to extract features from the input level. Low-level features are extracted from the previous level by DyDCNv2. High-level features are extracted from the next level by DyDCNv2. Then all features are weighted and summed.

The task-aware attention mechanism incorporates a dynamic differentiable activation function. Initially, it performs average pooling on the input features to reduce the number of channels. Subsequently, a fully connected layer is employed to derive dynamic parameters that can be adjusted based on the mean and standard deviation of the input features. Finally, the max function implements the dynamic activation function. 

Multi-attention mechanisms can be sequentially arranged and employed. Under the premise of considering computational costs, this study uses a separate set of multi-attention mechanism modules to optimize the feature expression ability of occluded targets in complex scenes and improve overall detection performance.

### 2.5. Loss Function

The effectiveness of object detection is partially reliant upon the design of the bounding-box loss function. Within the YOLOv7-tiny algorithm model, the CIoU Loss [43] serves as the designated bounding-box loss function. This loss function primarily considers parameters such as the overlapping area, center distance, and aspect ratio between the predicted box and the ground truth box. The equation is as stated:

(11) $$L_{CIoU}=L_{IoU}+\frac{\rho^{2}(b^{pr},b^{gt})}{c^{2}}+\alpha v$$

$L_{IoU}$ is utilized to quantify the extent of overlap between the predicted box and the true box in object-detection tasks. $\rho^{2}(b^{pr},b^{gt})$ denotes the Euclidean distance between the center points of the predicted box and the true box. c represents the diagonal distance between the smallest outer box of the wrapped predicted box and the true box. α is the weighting factor employed to adjust the balance ratio, and v is the parameter employed to assess the consistency of aspect ratio when the center points overlap. CIoU Loss, being a monotonically focused loss function, is often stable. However, when the aspect ratio of the predicted box and the true box is identical, αv vanishes, impeding model optimization and resulting in sluggish convergence of the loss function. 

This study introduces the WIoU Loss presented by Tong et al. [44] as a strategy to augment algorithmic performance and enhance detection accuracy. The loss function incorporates a dual-distance attention mechanism and a dynamic non-monotonic frequency modulation coefficient. The equations are as stated:

(12) $$L_{WIoUv3}=r\cdot R_{WIoU}L_{IoU}$$

(13) $$R_{WIoU}=\exp\left(\frac{\rho^{2}\left(b^{pr},b^{gt}\right)}{c^{2}}\right)$$

Among them, r is the dynamic nonmonotonic frequency modulation coefficient, $R_{WIoU}\in \left[1,e\right)$ is used to amplify the $L_{IoU}$ of ordinary quality anchor boxes, and $L_{IoU}\in$ [0,1] is used to reduce the $R_{WIoU}$ of high-quality anchor boxes. WIoU Loss with dynamic non-monotonic focusing mechanism dynamically allocates gradient gain for different quality image targets, further improving the accuracy and generalization ability of sensing.

### 2.6. PDT-YOLO Algorithm

**Figure 5:**
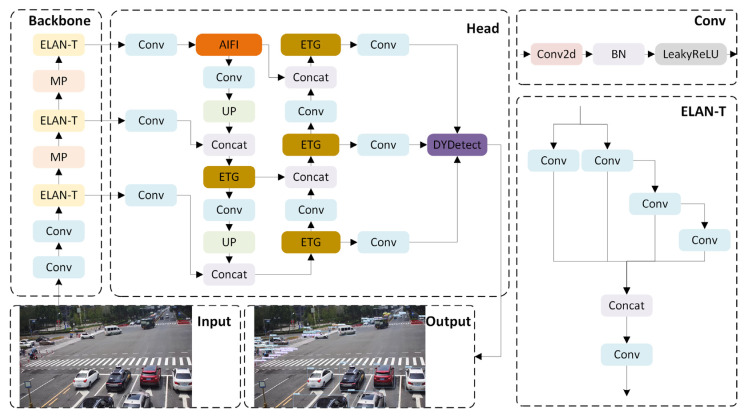
Model structure of PDT-YOLO algorithm.

This work presents a roadside object-detection algorithm for multiscale and occluded targets, based on the above-mentioned improvement strategy. Figure 5 displays the network architecture of the improved algorithm.

The backbone network plays a pivotal role in extracting features from input images and propagating these multi-layered feature representations to the subsequent head network. In the pursuit of lightweight deployment for roadside target detection models and the refinement of detection accuracy for small targets, we exclude 20 × 20 feature layers while augmenting the extraction process with 160 × 160 feature layers. Additionally, to enhance the extraction of more sophisticated and meaningful semantic features, a transformer-based AIFI module is introduced within the 40 × 40 feature layer. In the neck section, we substitute the original ELAN-T structure with the ETG structure to enhance the expressive capacity of image features without compromising the model’s parameter count or detection speed. The integrated multi-attention mechanism module in Detect enhances the model’s ability to process features in a more detailed manner, which allows the model to effectively concentrate on occluded target areas and reduce interference from the background. Moreover, by replacing the original CIoU Loss function with the WIoU Loss, the algorithm’s adaptability across diverse situations is enhanced, thereby amplifying the overall detection performance of the model.

## 3. Experimental Results and Analysis

### 3.1. Roadside Dataset 

In 2022, the Institute for AI Industry Research (AIR) of Tsinghua University released the DAIR-V2X dataset, a large-scale multi-modal multi-view object-detection dataset for research on vehicle-infrastructure cooperative autonomous driving relying on the high-level autonomous driving demonstration zone in Beijing. The DAIR-V2X dataset comprises a total of 71,254 sets of point cloud and image spatiotemporal synchronization data and annotation information. DAIR-V2X-C is a subset of DAIR-V2X, specifically designed for vehicle road collaborative perception, including 12,424 sets of point cloud and image spatiotemporal synchronization data and annotation information.

**Figure 6:**
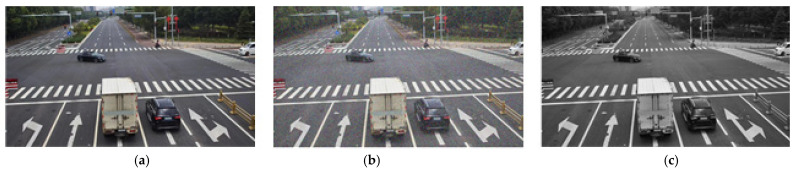
Enhancement results of a randomly selected image. (**a**) Origin image; (**b**) Image with random noise; (**c**) Image with random grayscale.

After statistical analysis of the dataset, due to the imbalance of data categories, random noise, and random grayscale are used to process the original image for data augmentation. Moreover, certain images underwent re-annotation procedures to maintain annotations across eight distinct categories: motorcyclist, traffic cone, pedestrian, cyclist, car, truck, van, and bus. The enhancement results of one picture in the DAIR-V2X-C dataset are shown in Figure 6.

**Figure 7:**
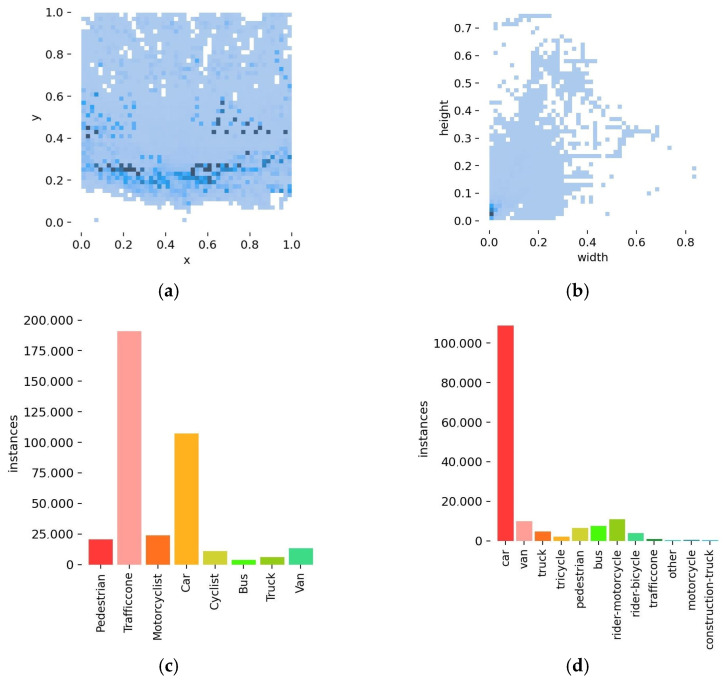
The visualization results of the enhanced DAIR-V2X-C dataset and IVODC dataset. (**a**) The distribution of the center position of the detection target of the DAIR-V2X-C dataset; (**b**) The distribution of the width and height of the label box for detecting targets of the DAIR-V2X-C dataset; (**c**) The distribution of the category and label quantity of the DAIR-V2X-C dataset; (**d**) The distribution of the category and label quantity of the IVODC dataset. In (**a**,**b**), the color of the dots represents the quantity, which means that the darker the color, the greater the quantity.

The enhanced DAIR-V2X-C dataset is partitioned into two subsets through random allocation, comprising a training set of 13,182 images and a testing set of 1643 images. Visualization of the enhanced DAIR-V2X-C dataset is presented in Figure 7. The dataset exhibits a broad distribution of object scales, with a notable prevalence of small objects.

The dataset used for generalization verification comes from the Intelligent V2X Open Dataset Challenge, jointly organized by the IMT-2020 (5G) Promotion Group Cellular Connected Vehicles (C-V2X) Working Group, China Association of Automobile Manufacturers and other organizations. The dataset includes 16,000 frames of image data and 8000 frames of LiDAR data. After assessment of the dataset, 84 frames of blank annotation information and corresponding image data are deleted, and 12 types of annotation information are merged and processed into 8 categories.

### 3.2. Experimental Environment and Evaluation Metrics

This study utilized the Ubuntu 20.04 operating system along with an i5-13600KF CPU and an NVIDIA GeForce RTX 3060 GPU. The programming language Python 3.8 was employed, with PyTorch 1.10 serving as the designated deep learning framework. To ensure the fidelity of the training outcomes, uniform training parameters were adopted across all algorithms. The model training parameters were configured as follows: a batch size of 8, a learning rate of 0.01, and a momentum of 0.937.

This article employs mean precision mAP50 and mean precision mAP50:95 as metrics to measure detection accuracy. Floating-point operations (GFLOPs), parameter counts (Params), and detection frames per second (FPS) are considered indicative measures of both efficiency and real-time performance. Additionally, the size of the model weight (Size) is utilized to assess the model’s suitability for deployment on edge devices.

(14) $$AP50=\frac{1}{n}\sum_{i=1}^{n}P_{i}^{IoU=0.5}\left(R_{i}^{IoU=0.5}\right)$$

(15) $$AP50:95=\frac{1}{10}\left(AP50+AP55+\cdots +AP95\right)$$

(16) $$mAP=\frac{1}{n}\sum_{j=1}^{n}AP\left(j\right)$$

P represents the accuracy rate, which is the ratio of successfully predicted positive samples to all predicted positive samples. R represents the recall rate, which is the ratio of correctly predicted positive samples to all real positive samples. AP50 refers to the mean average precision (AP) across all categories when the intersection over union (IoU) threshold is set at 50%. AP50:95 reflects the average AP values as the IoU threshold increases from 50% to 95% in increments of 5% and mAP stands for mean average precision and is the average accuracy calculated for each category.

### 3.3. Experimental Analysis

#### 3.3.1. Feature Fusion Network Improvement Experiment

To verify the effectiveness of the improved feature fusion network, separate experiments are conducted and compared with the original benchmark. The Tiny-P experiment refers to removing the input of the 20 × 20 feature layer from the neck network and adding the input of the 160 × 160 feature layer. The Tiny-AIFI experiment introduces a transformer-based AIFI module in the feature layer of 40 × 40. Table 1 presents the detailed results.

Table 1 shows that compared to the original YOLOv7-tiny algorithm model, the Tiny-P algorithm model improved by about 3.8% in mAP50 and 7.2% in mAP50:95. The number of parameters, weight, and detection speed have slightly decreased, while the GFLOPs has increased. Tiny-AIFI increased by approximately 0.4% in mAP50 and 2.8% in mAP50:95. The number of parameters, weight, GFLOPs, and detection speed have all slightly decreased. 

The results indicate that the above methods can effectively enhance the extraction of more informative and meaningful semantic features that the feature map has a smaller receptive field from the 160 × 160 feature layer and richer semantic concepts from the AIFI module. After multi-scale fusion, it can better learn object features, enhance the capture ability of the network for multi-scale objects, and improve the object-detection effect.

#### 3.3.2. ETG Module Experiment

To better evaluate the effectiveness of the ETG module in improving the detection accuracy of the YOLOv7-tiny algorithm model, this paper conducts experiments by introducing different numbers of GSConvs into the original ELAN-T structure of the YOLOv7-tiny algorithm. The modified models are named E-GS2 (introducing two GSConv modules) and E-GS3 (introducing three GSConv modules). Table 2 lists the results of experimental validation.

Table 2 shows that if only the GSConv module is introduced into the ELAN-T structure of the head part of the algorithm, as the number of GSConv modules increases, it can lighten the network structure, but at the same time, the detection performance of the algorithm will decrease. The results of the ETG module in this study reveal its efficacy in enhancing the detection accuracy of the model, though the parameter count has slightly increased. The structure of the ETG module employs two branches: one dedicated to preserving texture and background features of the image via 1 × 1 convolutions, while the other employs cascaded GSConv modules utilizing depth-wise separable convolution to optimize feature extraction, thereby facilitating more efficient information exchange among features within the neck. Among them, the most significant improvement is in mAP50:95, which increases by approximately 3.6%.

#### 3.3.3. Multi-Attention Mechanism Module Experiment

We added a DYDetect module based on multi-attention mechanisms to the feature fusion region of the original network and compared its performance with the original network. In order to guarantee the experiment’s efficacy, all data results have passed a minimum of 10 training and validation experiments. DYDetect makes the importance of various feature levels adaptive to the input, applies attention to each occluded object’s spatial location, and adaptively aggregates multiple feature levels together for learning a more discriminative representation which can focus on utilizing the visible information of occluded objects while ignoring the features of occluded parts. The experimental results are shown in Table 3, where mAP50 increased by 0.7% and mAP50:95 increased by 5.3%. In addition, the parameters, computational complexity, and weight of the model have all decreased. The result proves that the DYDetect model optimizes the feature-extraction ability of the original model, further improving the detection capability of the algorithm.

#### 3.3.4. Loss Function Experiment

This study conducts a comparison between the YOLOv7-tiny algorithm model and the YOLOv7-tiny enhanced Focal-EIoU Loss [45], MPDIoU Loss [46], and WIoU Loss. The purpose is to assess the efficacy of utilizing WIoU Loss in place of CIoU Loss. The precise outcomes are displayed in Table 4.

In Table 4, compared with the original YOLOv7-tiny algorithm model, the parameter count, GFLOPS, and detection speed of the Tiny-Focal-EIoU, Tiny-MPDIoU, and Tiny-WIoU algorithm remain unchanged, and the algorithm model has improved on mAP50 and mAP50:95. Among them, the Tiny-WIoU model outperforms the other models, increased by 0.3% on mAP50 and 3.2% on mAP50:95, which proves the WIoU model is effective for algorithm improvement. WIoU reduces the competitiveness of common high-quality anchor boxes while also reducing the harmful gradients generated by low-quality anchor boxes such as background noise, making the algorithm more focused on ordinary quality anchor boxes such as large-scale targets, small-scale targets, and occluded targets, thereby improving overall performance.

### 3.4. Overall Analysis

#### 3.4.1. Ablation Experiments

In this study, four improvements are proposed: feature fusion layer reconstruction, ETG module, DYDetect, and WIoU loss function. Under the same experimental setup, different ablation experiments were conducted on DAIR-V2X-C to verify the effectiveness of these four improved methods. The results of the ablation experiment are shown in Table 5. Experiment 1 is the original YOLOv7-tiny, while Experiment 2 reconstructs the feature fusion layer based on the original YOLOv7-tiny. Experiment 3 replaces the ELAN-T module with an ETG module based on the previous experiment, Experiment 4 integrates multiple attention modules into the detection head, and Experiment 5 introduces WIoU Loss based on the previous experiment.

Table 5 shows that, compared with the YOLOv7-tiny algorithm model, the PDT-YOLO model that integrates four improved methods outperforms the YOLOv7-tiny algorithm model; mAP50 increased by 4.6%, mAP50:95 increased by 12.8%, and the parameters and weight of the model remain unchanged. Although the detection speed has declined, it still meets the real-time requirements. 

**Figure 8:**
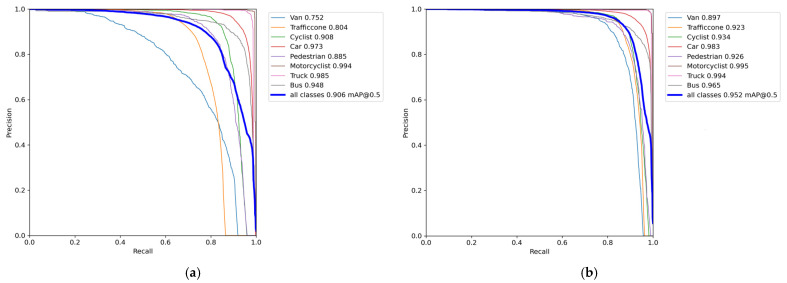
PR-Cure comparison. (**a**) YOLOv7-tiny; (**b**) PDT-YOLO.

PR-Cure is shown in Figure 8. From the figure, it can be seen that compared with the original YOLOv7-tiny, our algorithm effectively improves the detection accuracy of various categories on the DAIR-V2X-C dataset. Especially, van, traffic cone, and pedestrian each improved by 14.5%, 11.9%, and 4.1%, proving that the improved algorithm has better improvement in multi-scale object-detection ability and detection ability for dense small targets.

#### 3.4.2. Comparison Experiments

To evaluate the effectiveness of the network model proposed in this paper, the PDT-YOLO network is compared with mainstream algorithms in the field of object detection, such as SSD, YOLOXs, YOLOv5s, YOLOv7-Tiny, and YOLOv8s. In addition, we also compare it with multi-scale object-detection algorithms [28,29] and occlusion object-detection algorithms [35]. The detection outcomes of the comparative trials are presented in Table 6.

In comparison to SSD, the YOLO series has made substantial enhancements in terms of mAP50, and mAP50:95. Compared with the original YOLOv7-tiny algorithm, our algorithm has improved by 4.6% and 12.8% on mAP50 and mAP50:95, respectively, with little change in weight and parameter counts. Compared to the latest YOLOv8s algorithm, it has improved by 3.3% and 0.7% on mAP50 and mAP50:95, respectively, reduced parameter counts by 45%, and reduced model weight by 43.6%. Compared with the multi-scale object-detection [28,29] and occlusion object-detection [35] algorithms, algorithms with more complex network models, the proposed network using the lightweight network as the base model, also showed certain advantages in detection accuracy, weight, and parameter counts.

From the experimental results, it can be seen that the improved network not only improves the detection accuracy of the model but also reduces the number of parameters and the weight of the model, making it well-suited for complex roadside target detection tasks under resource-limited conditions.

**Figure 9:**
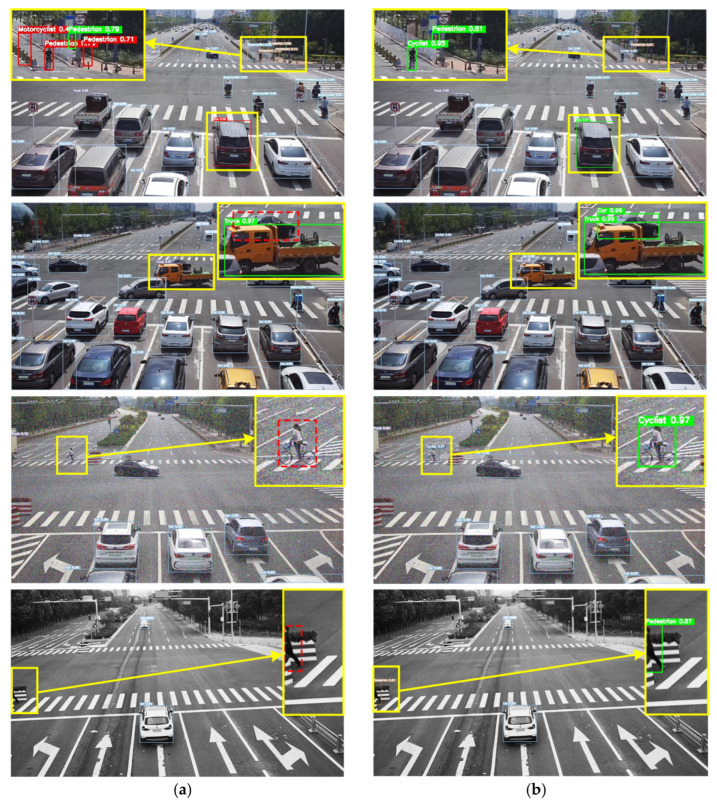
Visualization of algorithm model detection results on the DAIR-V2X-C dataset. (**a**) YOLOv7-tiny; (**b**) PDT-YOLO. All of the key areas with yellow boxes are enlarged and displayed. The red solid line indicates false detection, the red dashed line indicates missed detection and the green line indicates correct detection.

Figure 9 shows the visualization results of the benchmark algorithm and our algorithm. The objects depicted in the roadside photos exhibit different scales and types, and even objects of the same type have significant differences in scale. For the convenience of comparison, we will mark the key areas with yellow boxes and enlarge them. The red solid line indicates false detection, the red dashed line indicates missed detection and the green line indicates correct detection. Compared horizontally, YOLOv7-tiny has many false positives and missed detections. In the first row of images, it can be seen that under normal conditions, the proposed method accurately detected multi-scale objects, while YOLOv7-tiny shows that small and distant targets such as cyclists and infrastructure are mistakenly detected as pedestrians, while large nearby targets such as the car is identified as a bus. YOLOv7-tiny misses the detection of the car that is obstructed by a truck in the second row of the image. In the third row of the image with random noise, when the decrease in image clarity leads to increasing difficulty in detecting, YOLOv7-tiny misses the detection of the cyclist, while PDT-YOLO accurately detects all the objects in the image. In the fourth row of the image’s random grayscale, when the image is disturbed by illumination changes, YOLOv7-tiny misses the detection of pedestrians, while PDT-YOLO accurately detects all the objects in the image. In contrast, the algorithm proposed in this article has better detection performance compared to the original benchmark algorithm in complex traffic scenes such as multi-scale targets and occluded targets.

#### 3.4.3. Generalization Experiments

In order to further verify the stability and generalization of the improved algorithm in this paper, the algorithm studied in this paper will be re-validated with the other three algorithms in the IVODC dataset.

From Table 7, it can be seen that compared to the original YOLOv7-tiny, both mAP50 and mAP50:90 have been improved. At the same time, the weight and parameter quantity remain basically unchanged. Compared with the latest algorithm YOLOv8s, the PDT algorithm model has significantly reduced the weight and parameter quantity and has greatly improved the detection accuracy. PDT-YOLO achieves higher accuracy, effectively compresses the model weight, and improves the efficiency of model deployment in edge devices.

**Figure 10:**
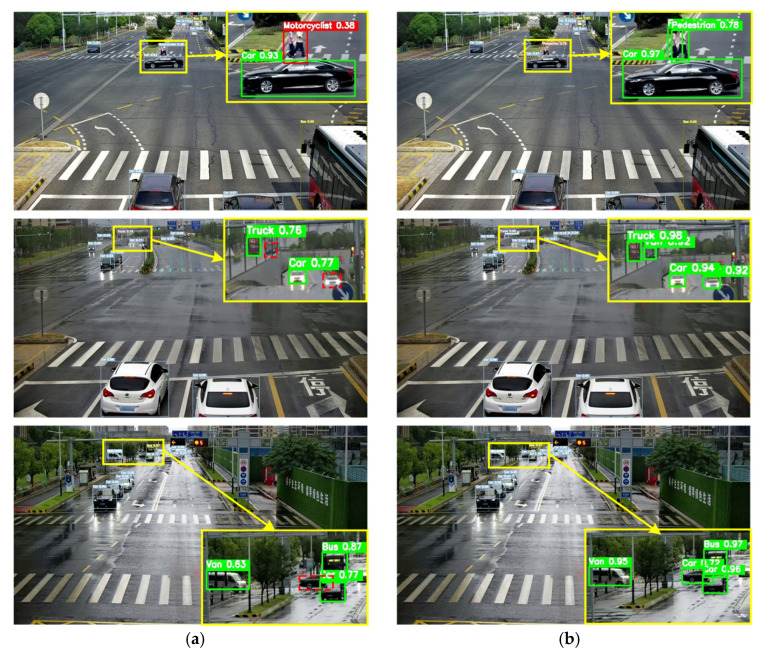
Visualization of algorithm model detection results on the IVODC dataset. (**a**) YOLOv7-tiny; (**b**) PDT-YOLO. All of the key areas with yellow boxes are enlarged and displayed. The red solid line indicates false detection, the red dashed line indicates missed detection and the green line indicates correct detection.

Visualize benchmark algorithms YOLOv7-tiny and PDT-YOLO on the IVODC dataset in Figure 10. The IVODC dataset mainly includes rainy traffic scenes in which the images obtained by roadside cameras are less clear compared to sunny days, and due to ground reflection, false positives are more likely to occur. For the convenience of comparison, we will mark the key areas with yellow boxes and enlarge them. The red solid line indicates false detection, the red dashed line indicates missed detection and the green line indicates correct detection. From the first row of Figure 10, it can be seen that under sunny conditions, the benchmark algorithm recognizes two pedestrians behind a car as a motorcyclist. In the second row of cloudy and rainy weather conditions, a vehicle with lights running out from the underground passage in the distance is missed detection and a car that is blocked by other vehicles in dense conditions is missed detection in the third row. From the three comparison graphs above, PDT-YOLO has a stronger perception ability and lower missed detection rate compared to benchmark algorithms, whether in dense and occluded target environments or traffic environments such as rainy and weak light. Sending the data identified by the algorithm in this article to autonomous or regular vehicles for safety warning through the communication module can improve driving safety in rainy conditions.

### 3.5. Validation on Real Traffic Scene

**Figure 11:**
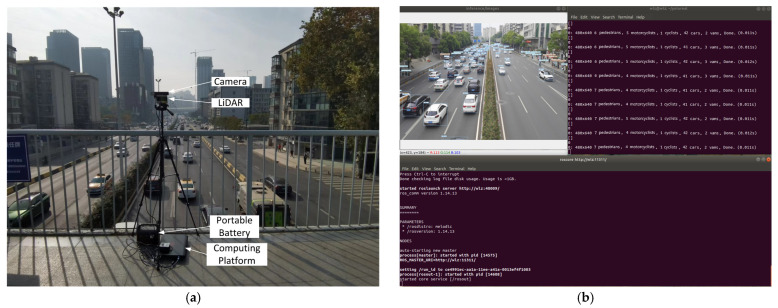
Roadside object-detection system deployment diagram. (**a**) Composition of roadside object-detection system; (**b**) Real-time detection interface based on ROS system.

A roadside target detection system is deployed on a pedestrian bridge in Wuhan for actual performance verification. The system includes a roadside computing platform, cameras, LiDAR, a portable battery, and a visualization interface. The roadside computing platform is configured with the environment of Ubuntu 20.04, Python 3.8, CUDA11.3, PyTorch 1.10, and ROS system which displays a real-time detection visualization interface. A roadside camera is an industrial camera used to collect roadside video data. The system deployment and real-time detection visualization interface are shown in Figure 11. The visualization interface can display the number and type of targets in each frame of the image, as well as the inference time of each frame of the image. From Figure 11b, it can be seen that there are 7 pedestrians, 4 motorcyclists, 1 cyclist, 42 vehicles, and 2 vans in the current environment at a speed of 90 frames per second (FPS) approximately.

**Figure 12:**
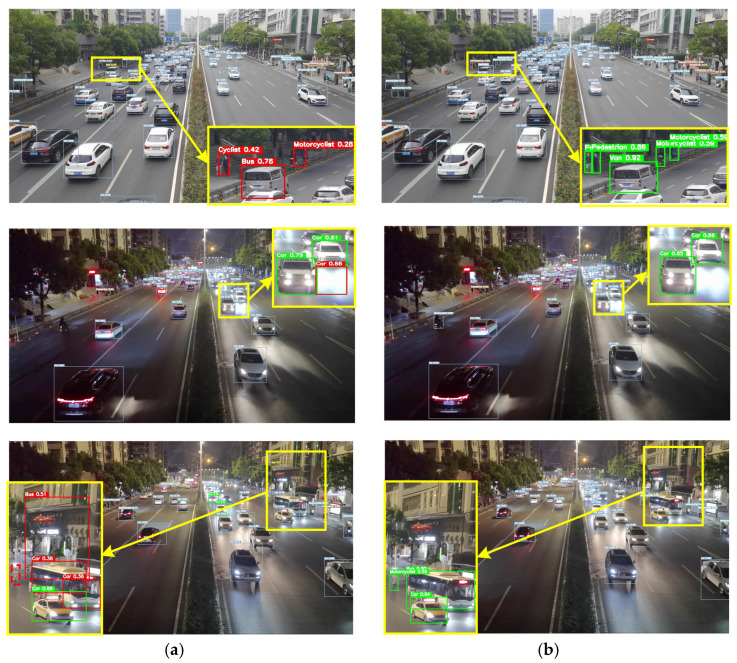
Visualization of algorithm model detection results on actual roads. (**a**) YOLOv7-tiny; (**b**) PDT-YOLO. All of the key areas with yellow boxes are enlarged and displayed. The red solid line indicates false detection, the red dashed line indicates missed detection and the green line indicates correct detection.

The detection results of the roadside target detection system based on actual deployment are shown in Figure 12. The images that need to be detected are day and night scenes during traffic congestion. For the convenience of comparison, we will mark the key areas with yellow boxes and enlarge them. The red solid line indicates false detection, the red dashed line indicates missed detection and the green line indicates correct detection. Compared with the benchmark algorithm, the PDT-YOLO algorithm can accurately detect dense small-scale targets such as first-ranked people and vehicles. In the second and third rows, in complex environments such as low light, the YOLOv7-tiny algorithm mistakenly detects ground-reflected light as vehicles, the house on the roadside as a bus, and buses as multiple vehicles, and misses small targets such as motorcycles. The above results indicate that the algorithm proposed in this paper can achieve accurate detection of traffic participants from an actual roadside perspective, and performs better than the benchmark algorithm in low-light environments, occlusion, and multi-scale targets, making it more suitable for deployment on the roadside.

## 4. Discussion

In recent years, intelligent roadside perception systems have always been hot spots of scientific research [4,5,6,7,8,9,10]. Some scholars [24,25] have adopted the deep learning algorithm to address several challenges, including weak multi-scale object perception ability, the high missed detection rate of occluded targets, and difficulties in model deployment.

Target sizes vary in roadside perception scenarios. In this case, extracting target features solely through a single scale is extremely difficult. To address this, the integration of multi-scale context information becomes imperative, as it can enhance detection performance. Researchers have explored various approaches to extract contextual information across different scales. These include employing diverse convolutional kernels to extract features at multiple scales [26], cascading hierarchical structures to propagate information [27,30], such as feature pyramid frameworks, increasing the number of detection heads [28,29], and other techniques aimed at aggregating information from input images. In our study, we reconstruct the feature pyramid structure by adding the 160 × 160 feature map while eliminating the 20 × 20 feature map. This adjustment prioritizes the processing of feature maps sized 160 × 160, 80 × 80, and 40 × 40 to effectively combine contextual information for smaller objects. Moreover, higher-level features are derived from lower-level features containing rich semantic information about objects within the image. Such high-level features, characterized by richer semantic concepts, facilitate the discernment of connections between conceptual entities within the image, aiding detection and recognition by subsequent modules. Intra-scale interactions among lower-level features are unnecessary due to their lack of semantic concepts, which may risk duplicating or confounding interactions with high-level features. The introduction of the AIFI module performs intra-scale interaction on the 40 × 40 high-level feature map to extract deeper features. Based on Table 5, our feature fusion network enhances the extraction ability of high-level and low-level features, effectively improving the detection accuracy of the model. Meanwhile, due to the removal of the original 20 × 20 feature map, the parameters and weight of the model have also been reduced.

Due to the high density of objects and low camera angle for monitoring urban traffic, occlusion will lead to false detections of occluded objects. Some methods have been proposed to solve this problem. Attention maps that guide the learning of visible parts [33], such as pixel-by-pixel and channel-by-channel attention maps, can learn robust feature representations to focus on relevant information. Special-designed occlusion-aware loss functions [35,36] can automatically increase the weight of healthy samples and reduce the weight of false positives. Post-processing techniques such as non-maximum suppression (NMS) [34] with specially designed thresholding can refine the detection capability. We use a method of data augmentation using random noise and random grayscale to enhance the dataset and improve the model’s ability to generalize to different scenarios such as partial occlusion, object overlap, and cluttered backgrounds. We integrate a multi-attention mechanism concluding the scale-ware attention, the spatial-aware attention module, and the task-aware attention module. The multi-attention mechanism makes the importance of various feature levels adaptive to the input, applies attention to each occluded object’s spatial location, and adaptively aggregates multiple feature levels together for learning a more discriminative representation which can focus on utilizing the visible information of occluded objects while ignoring the features of occluded parts. WIoU reduces the competitiveness of common high-quality anchor boxes while also reducing the harmful gradients generated by low-quality anchor boxes such as background noise, making the algorithm more focused on ordinary quality anchor boxes such as large-scale targets, small-scale targets, and occluded targets, improving overall performance.

We introduced the GSConv module for efficient layer aggregation and constructed the ETG module. GSConv consists of a standard convolution module, a depth-wise separable convolution module, and a Shuffle. Compared to standard convolutional kernels, GSCONV may not have strong feature extraction capabilities, but it can optimize model parameters. The ETG module is based on a stacked structure design, two branches are used to preserve the texture and background features of the image using 1 × 1 convolution, while the other two branches use two cascaded GSConv modules to improve feature fusion and optimize the model parameters, effectively reducing network complexity.

Our experimental results demonstrated that, compared to YOLOv7-tiny, PDT-YOLO improves mAP50 and mAP50:95 by 4.6% and 12.8% while maintaining comparable parameter count and weight. Additionally, PDT-YOLO outperforms several mainstream algorithms, multi-scale target detection, and occluded target detection algorithms, in terms of mAP50, mAP50:90, parameter count, and weight on the DAIR-V2X-C dataset. Moreover, the generalization and effectiveness of the algorithm were validated through testing on the IVODC dataset, showing its robust performance even in challenging environmental conditions such as cloudy and rainy weather. Real-world deployment on actual roads using ROS systems further confirmed the algorithm’s capability to detect multi-scale and occluded targets at a speed of 90, meeting the demands of edge target detection in complex traffic scenarios.

In conclusion, the PDT-YOLO algorithm presents a solution for improving the performance of intelligent roadside perception systems. Its effectiveness in addressing challenges of weak multi-scale object perception and high missed detection rate of occluded targets highlights its potential for real-world deployment for further research and development in the field of intelligent transportation systems.

## 5. Conclusions

This article proposes a roadside object-detection algorithm for multiscale and occluded targets algorithm called PDT-YOLO and conducts ablation and comparison experiments on the DAIR-V2X-C dataset, generalization experiments on the IVODC dataset, and deployment verification on an actual traffic environment. Experimental results have shown that the PDT-YOLO outperformed mainstream algorithms in terms of accuracy, with small model size, minimal parameter count, and fast inference speed, meeting the demands of edge target detection in complex traffic scenarios.

However, the algorithm does not involve low visibility of cameras under extreme weather conditions such as heavy snow, so the algorithm still has certain limitations. Future work will optimize the model to enhance its robustness, accuracy, and speed, thus ensuring its applicability across a wider range of environmental conditions.

## Figures and Tables

**Table 1:** Feature fusion network experiment verification.

Method	Params (M)	GFLOPs	Size	mAP@0.5 (%)	mAP@0.5:0.95 (%)	FPS
YOLOv7-tiny	6.0	13.2	12.3	90.6	62.8	156
Tiny-P	5.8	30.2	12.1	94.4	70	123
Tiny-AIFI	5.7	12.8	11.6	91	65.6	131

**Table 2:** ETG module experiment verification.

Method	Params (M)	GFLOPs	Size	mAP@0.5 (%)	mAP@0.5:0.95 (%)	FPS
YOLOv7-Tiny	6.0	13.2	12.3	90.6	62.8	156
Tiny-E-GS2	5.8	13.0	12.0	89.9	62	149
Tiny-E-GS3	5.7	12.7	11.8	89.4	61.7	138
Tiny-ETG	6.1	13.4	13.2	91.3	66.4	125

**Table 3:** Multi-attention mechanism module experiment verification.

Method	Params (M)	GFLOPs	Size	mAP@0.5 (%)	mAP@0.5:0.95 (%)	FPS
YOLOv7-tiny	6.0	13.2	12.3	90.6	62.8	156
Tiny-DYDetect	5.9	12.9	12.2	91.9	68.1	101

**Table 4:** Comparison of experiments of the loss function.

Method	Params (M)	GFLOPs	Size	mAP@0.5 (%)	mAP@0.5:0.95 (%)	FPS
YOLOv7-tiny (CIoU)	6.0	13.2	12.3	90.6	62.8	156
Tiny-Focal-EIoU	6.0	13.2	12.3	90.7	65.5	157
Tiny-MPDIoU	6.0	13.2	12.3	90.7	65.8	157
Tiny-WIoU	6.0	13.2	12.3	90.9	66	161

**Table 5:** Comparison of ablation experiments.

Method	P-AIFI	ETG	DYHead	WIoU	Params (M)	Size	mAP@0.5 (%)	mAP@0.5:0.95 (%)	FPS
1					6.0	12.3	90.6	62.8	156
2	√				5.6	11.7	94.1	71.3	128
3	√	√			6.1	12.8	94.3	72.2	101
4	√	√	√		6.1	12.7	94.8	75	98
5	√	√	√	√	6.1	12.7	95.2	75.6	98

**Table 6:** Performance comparison of algorithm models on the DAIR-V2X-C dataset.

Method	Params (M)	Size	mAP@0.5 (%)	mAP@0.5:0.95 (%)
SSD	26.3	98.7	71.7	44.7
YOLOXs	54.2	36.0	85.1	55.2
YOLOv5s	7.0	14.4	88.1	63.1
YOLOv7-tiny	6.0	12.3	90.6	62.8
YOLOv7	36.5	74.8	94.5	71.9
YOLOv8s	11.1	22.5	91.9	74.9
Shen [28]	62.5	234	91.1	63.3
Ju [29]	63.7	245.3	92.4	64.7
Li [35]	50.4	179.4	91.6	66.2
PDT-YOLO	6.1	12.7	95.2	75.6

**Table 7:** Performance comparison of mainstream algorithm models on the IVODC dataset.

Method	Params (M)	Size	mAP@0.5 (%)	mAP@0.5:0.95 (%)
YOLOv7-tiny	6.0	12.4	76.4	54.7
YOLOv7	36.5	74.9	91.7	63.7
YOLOv8s	11.1	22.6	80.5	60.3
PDT-YOLO	6.1	12.5	92.1	65.8

## Data Availability

Data are contained within the article.
